# Characterization of immunomodulatory activity of eIF4A protein

**Published:** 2011-01-10

**Authors:** Mourad Barhoumi, Olga Koutsoni, Ikram Guizani, Eleni Dotsika

**Affiliations:** 1 Laboratoire d’Epidémiologie et d’Ecologie Parasitaire, Institut Pasteur de Tunis, 1002 Tunis-Belvedère, Tunisia; 2 Laboratory of Cellular Immunology, Departement of Microbiology, Hellenic Pasteur Institute, Athens 11521, Greece

## 

*Leishmania* LeIF antigen, homologous to eukaryotic initiation factor eIF4A, was originally described as a Th1-type natural adjuvant and as an antigen that induces an IL-12-mediated Th1 response in the peripheral blood mononuclear cells (PBMC) of leishmaniasis patients. We showed previously that the induction of cytokines in monocytes of healthy subjects is not unique to the *Leishmania* protein. Indeed, 5 homologous proteins DEAD box in mammals and yeast were also able to induce the secretion of cytokines in monocytes of healthy subjects. In this study we aimed to validate eIF4A protein as a natural adjuvant. To achieve this objective, LeIF, eIF4A from mouse (MeIF4A) and Yeast (YeIF4A) were expressed and purified. The purified proteins were assessed for their ability to induce maturation of bone marrow-derived dendritic cells (BMDC). Their effect on DCs and their monocytic precursors in the peritoneal cavity of mice were analyzed. Our data showed that eIF4A proteins were able to activate BMDC to express co-stimulatory molecules and to produce IL-12p40/p70 and iNOS *in vitro*. Furthermore, eIF4A proteins induced inflammation in the peritoneal cavity of BALB/c mice similar to the well known adjuvant Alum. Indeed, injection of eIF4A proteins in combination with OVA protein induced a rapid recruitment into the peritoneal cavity of Ly6C^high^/CD11b^+^ inflammatory monocytes, Ly6G^high^/CDb^+^ neutrophils and myeloid dendritic cells (MHC IIhigh-CD11c+). This study highlights the adjuvant activity of eIF4A protein and suggests that the immunomodulatory properties of eIF4A could be exploited in vaccination or immunotherapy protocols.

Supported by grants from MESRST-Tunisia (LR00SP04), UNICEF/UNDP/WorldBank/WHO special programme TDR (A30134) and a Fellowship from the International Network Institut Pasteur.

